# Cellular insights into transposable elements in Alzheimer’s disease

**DOI:** 10.3389/fmolb.2025.1642599

**Published:** 2026-01-07

**Authors:** Vikas Kumar, Samuel Beck

**Affiliations:** 1 Center for Aging Research, Department of Dermatology, Chobanian & Avedisian School of Medicine, Boston University, Boston, MA, United States; 2 Department of Biology, College of Science, United Arab Emirates University, Al-Ain, United Arab Emirates

**Keywords:** Alzheimer disease, bioinformatics, differential expression and marker genes, single-cell, transposable elements

## Abstract

**Introduction:**

Alzheimer’s disease (AD) is a progressive neurodegenerative disorder affecting millions worldwide. While advances in single-cell technologies have elucidated cellular diversity and transcriptional changes in AD, the contribution of transposable elements (TEs) to disease pathogenesis remains poorly understood.

**Methods:**

We integrated published single-nucleus RNA sequencing data from 11 AD patients and 7 controls with chromatin accessibility profiles from ATAC-seq to map the cell type—specific landscape of TE expression and regulation.

**Results:**

We identified 508 differentially expressed TE loci, 84.3% of which were upregulated in AD, indicating widespread TE activation. TE dysregulation was most prominent in excitatory neurons (319 loci) and oligodendrocytes (165 loci), dominated by SINE (62.8%) and LINE (26.4%) elements. Several dysregulated TEs overlapped regulatory regions near key AD-associated genes including *DOC2A*, *ABCA7*, *PTK2B*, *IL34*, *ABCB9*, *PLD3*, and *TARDBP*.

**Discussion:**

These findings highlight cell-type-specific TE activation in AD and provide a foundation for investigating TE-mediated regulatory disruption and its therapeutic potential.

## Introduction

Alzheimer’s disease (AD) is the most prevalent neurodegenerative ailment affecting the elderly and has shown a sharp rise in prevalence over the past several decades. Alzheimer’s disease is characterized by progressive neurodegeneration that includes cognitive declines such as memory loss, diminished attention and language skills, and decreased ability to carry out daily tasks (Biamonti et al., 2021). Two defining pathological features of the AD brain are extracellular β-amyloid (Aβ) plaques and intracellular neurofibrillary tangles resulting from tau hyperphosphorylation. (Ittner and Götz, 2011; Biamonti et al., 2021). Additionally, AD also severely impairs the neurovascular unit (NVU), a tightly integrated network of neurons, glia, vascular cells, and pericytes responsible for maintaining brain homeostasis (Zlokovic, 2011).

Advances in single-cell and single-nucleus RNA sequencing (sc/snRNA-seq) have transformed the study of AD by enabling high-resolution profiling of cellular diversity and transcriptional states within the human brain. These approaches have identified numerous neuronal, glial, and immune cell subtypes that contribute to AD pathophysiology (Mathys et al., 2019; Lau et al., 2020; Morabito et al., 2020; Yang et al., 2022). Analyses of cortical and hippocampal regions in AD patients have revealed dysregulation of key biological processes such as angiogenesis, immune activation, synaptic signaling, and myelination, alongwith alterations in cellular composition (Lau et al., 2020). Importantly, expression of the major AD risk gene *APOE* varies across cell types, being reduced in oligodendrocyte progenitors and selected astrocyte subsets but strongly upregulated in a disease-associated microglial state (Grubman et al., 2019).

Accumulating evidence links tau pathology in AD to aberrant activation of transposable elements (TEs). RNA-sequencing of human postmortem brain tissue has revealed widespread upregulation of Long Interspersed Nuclear Element (LINE-1) and Endogenous Retroviral Elements (ERVs) in individuals with high neurofibrillary tangle burden (Guo et al., 2018). Transposable elements constitute a significant portion of the human genome, with Class I TEs (retrotransposons) including Human Endogenous Retroviruses (HERVs) (∼8%), Long Interspersed Nuclear Elements (LINEs) (∼21%), and Short Interspersed Nuclear Elements (SINEs) (∼13%), while Class II TEs (DNA transposons) account for ∼2% (Cosby et al., 2019). Previous studies in AD have primarily focused on LINE-1 and HERV activity, with increased LINE-1 RNA and ORF1p protein levels particularly evident in microglia linked to late-onset AD (Ravel-Godreuil et al., 2021; Evering et al., 2023). Elevated LINE-1 expression and ORF1p immunoreactivity in microglia were observed in late-onset Alzheimer’s disease (LOAD), correlating with disease-associated microglial morphology (Ravel-Godreuil et al., 2021; Evering et al., 2023). CRISPR-mediated activation of LINE-1 in iPSC-derived microglia resulted in disrupted morphology, impaired amyloid-β phagocytosis, and transcriptomic changes linked to antigen presentation, lipid metabolism, and AD-relevant genes, suggesting a key role for LINE-1 in LOAD pathogenesis (Roy et al., 2024).

Dysregulation of TEs can influence gene expression through insertional mutagenesis and regulatory disruption, cause genomic instability via DNA breaks, and exacerbate neuroinflammation through activation of innate immune pathways (Guo et al., 2018; De Cecco et al., 2019). Single-cell resolution of TE activity has transformed our understanding of their role in AD pathogenesis and established their potential as biomarkers and therapeutic targets. Despite recent advances, key questions remain: which brain cell populations are most vulnerable to TE activation, whether AD pathology selectively triggers specific TE subfamilies, and how dysregulated TE expression disrupts normal gene regulatory programs and cellular homeostasis. In this study, using the single-nuclei data, we show that TEs are differentially regulated, and their expression may impact disease progression in AD.

## Result

### TE expression during the AD progression

**FIGURE 1:**
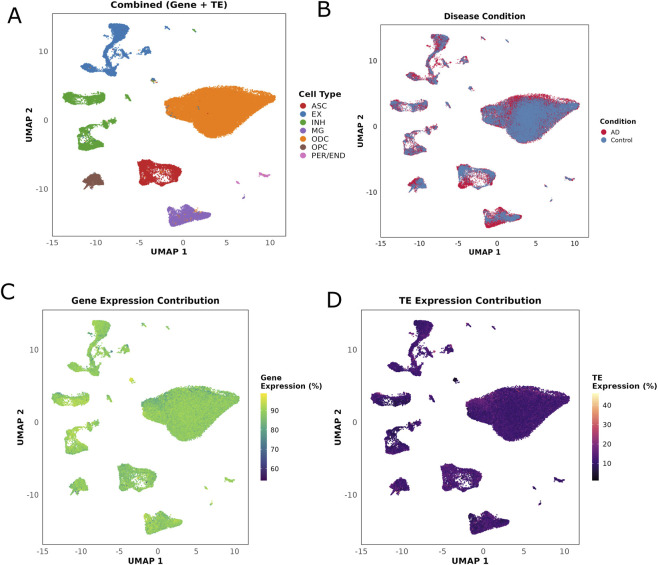
UMAP visualization of single-nucleus RNA-seq data revealing cell type distribution and transposable element expression patterns in Alzheimer’s disease brain tissue. **(A)** UMAP projection of 61,472 nuclei colored by major cell type annotations based on combined gene and transposable element (TE) expression. Eight distinct cell populations were identified: astrocytes (ASC), excitatory neurons (EX), inhibitory neurons (INH), microglia (MG), oligodendrocytes (ODC), oligodendrocyte precursor cells (OPC), and pericytes/endothelial cells (PER/END). **(B)** The same UMAP projection colored by disease condition, showing the distribution of nuclei from Alzheimer’s disease (AD, red) and control (blue) samples. **(C)** UMAP visualization showing the relative contribution of gene expression to total cellular transcripts. Color gradient represents the percentage of total transcipt counts derived from protein-coding genes. **(D)** UMAP visualization showing the relative contribution of TE expression to total cellular transcripts. Color gradient represents the percentage of UMI counts derived from transposable elements.

We analyzed published single-nucleus RNA-seq data from 11 individuals with AD and 7 age-matched controls (Morabito et al., 2021) with details provided in (Supplementary Table S1). Initially, the raw sequencing data were aligned to the human genome using CellRanger with default parameters (Zheng et al., 2017). The resulting BAM alignment files were later processed using SoloTE (Rodríguez-Quiroz and Valdebenito-Maturana, 2022) to generate matrices containing gene and TE expression for each cell. Later, we used Seurat (http://www.satijalab.org/seurat) (Hao et al., 2024) for the locus-specific TE matrices for data processing and clustering. The UMAP clustering plot generated by the Seurat was used to visualize the data in a two-dimensional subspace, revealing 34 distinct cell clusters (Supplementary Figure S1B). These 34 different clusters were further annotated as eight major clusters defining eight major cell-types of microglia, astrocytes, oligodendrocytes, oligodendrocyte precursor cells (OPCs), excitatory neurons, inhibitory neurons, endothelial cells (ECs), and pericytes matching the cellular group annotation of (Morabito et al., 2021) (Figure 1; Supplementary Figure S1A).

The additional UMAP plots with other covariates such as the age, sex, and disease condition presented similar clustering pattern with homogenous distribution pattern among the different clusters in UMAP (Figure 1; Supplementary Figure S1). UMAP projections colored by age and sex showed that cells were well intermixed across demographic groups, with no visible separation within or between clusters (Supplementary Figure S1C–D). When colored by disease condition, cells from AD and control samples were well-intermixed within each cell type cluster, indicating successful batch correction and absence of any major technical artifacts (Figure 1B). Analysis of relative expression contributions revealed that gene expression comprised 85%–90% of total transcript counts across all cell types (Figure 1C), whereas TE expression accounted for 10%–15% of cellular transcripts, with notable heterogeneity both within and between cell types (Figure 1D). While the mean TE contribution was relatively consistent across cell types (ranging from 11.02% to 13.33%), individual cells showed high variation in TE expression levels, with some cells exhibiting up to 40% TE-derived transcripts. This variability in TE expression is particularly evident in the oligodendrocyte and neuronal nuclei, suggesting dynamic regulation of TE activity that may be influenced by cellular state or disease processes rather than cell type identity alone (Figure 1; Supplementary Table S2). Nuclei distribution analysis across samples confirmed adequate representation of major cell types for differential expression analysis with oligodendrocytes showed the highest abundance (median >1,200 nuclei/sample) (Supplementary Table S2; Supplementary Figure S2). Cell type distributions were comparable between AD and control conditions, with most cell types showing median counts of 150–400 nuclei per sample, supporting the validity of our differential expression results across populations (Supplementary Table S2; Supplementary Figure S2).

### Differential expression results of genes and TE elements

**FIGURE 2:**
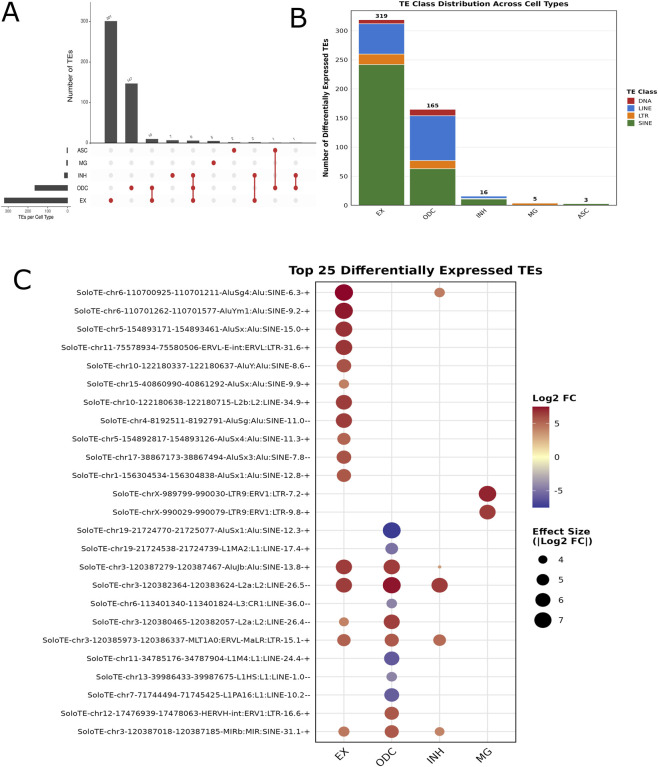
Differentially expressed transposable elements (TEs) across major brain cell types. **(A)** UpSet plot showing the total number of unique TEs identified in each major brain cell type, including oligodendrocytes (ODC), excitatory neurons (EX), inhibitory neurons (INH), astrocytes (ASC), and microglia (MG). The bar plot above represents the number of TEs detected in each unique or shared cell type set. **(B)** Bar plot summarizing the distribution of TE element types-DNA transposons, LINEs, LTRs, and SINEs—within each cell type. LINE and SINE elements were the most abundant across cell types, with distinct enrichment patterns. **(C)** Dot plot showing the top 25 differentially expressed TE markers across cell types. Dot size represents the proportion of cells expressing the TE, while color intensity reflects the average scaled expression. This visualization highlights cell-type-specific enrichment of TE activity, particularly in oligodendrocyte and excitatory neurons.

We identified 508 differentially expressed TE loci between normal and AD conditions after stringent filtering and adjustment for sample bias using sensitivity analysis (p < 0.05 and |log_2_FC| ≥ 2.0; see Methods for details) (Supplementary Table S3; Figure 2A). While larger cell clusters detected more total TEs as expected (Spearman ρ = 0.919, p = 0.003), normalization revealed that excitatory neurons showed the highest rate at ∼50 TEs per 1,000 cells, ∼11-times higher than oligodendrocytes (4.5 TEs/1,000 cells) despite being 6-times smaller. Effect sizes were independent of cluster size (r = −0.626, p = 0.258), with smaller clusters often showing stronger fold-changes than larger ones. Two cell types (OPC and pericytes/endothelial) showed zero dysregulated TEs despite containing 2,740 and 467 cells respectively. These patterns confirm that elevated TE dysregulation in excitatory neurons and oligodendrocytes reflects true AD-related biological differences rather than statistical artifacts from unequal cell numbers. In addition, the pseudobulk methodology effectively controlled for cluster size variation by testing at the sample level (n = 18) rather than cell level.

Most of these differentially expressed TEs showed cell-type-specific patterns, with minimal overlap between cell populations, 462 TEs (95.9%) were unique to single cell types while only 20 TEs (4.1%) were shared across multiple cell types as shown in (Figure 2A). This pronounced cell-type specificity suggests these locus-specific markers may play distinct regulatory roles in different cell types. Further classification revealed four major TE classes, including SINEs, LINEs, Long Terminal Repeats (LTRs), and DNA transposons (Figure 2B). Among these, SINEs were the most abundant, comprising 319 elements (62.8%), followed by LINEs with 134 elements (26.4%), LTRs with 37 elements (7.3%), and DNA transposons with 18 elements (3.5%). (Figure 2B). The top 25 most significantly DE TEs showed distinct expression patterns across cell types, with particularly strong differential expression observed in excitatory neurons (16 of top 25) and oligodendrocytes (8 of top 25) (Figure 2C). Our single-cell resolution of SINE/Alu, LINE, ERV and DNA transposon dysregulation corroborates previous bulk tissue studies that identified these same TE families in AD pathogenesis (Evering et al., 2023; Mustafin and Khusnutdinova, 2024) while extending these findings by revealing cell-type-specific activation patterns. The predominance of SINE and LINE elements, which together comprise 89.2% of our dysregulated TEs, suggests these retrotransposon families are central drivers of TE-mediated pathology in AD. Directional analysis revealed a predominant trend toward TE activation in AD, with 428 TEs (84.3%) showing upregulation and 80 TEs (15.7%) showing downregulation in AD samples compared to controls (Supplementary Table S3). This pattern was consistent across most cell types, with excitatory neurons showing 95.0% upregulated TEs (303/319), inhibitory neurons 75.0% (12/16), microglia 80.0% (4/5), astrocytes 66.7% (2/3), and oligodendrocytes showing a more balanced pattern with 64.8% upregulated (107/165) and 35.2% downregulated (58/165).

### Cell-type-specific and chromosomal TE dysregulation in AD

Analysis of the chromosomal distribution of the 508 robust DE TEs revealed non-random genomic patterns with distinct chromosomal hotspots (Supplementary Figure S3; Supplementary Table S3). Across all chromosomes, upregulation was the predominant pattern, accounting for 77.4% of DE TEs, with chromosome 3 harboring the highest absolute number (∼50 TEs), followed by chromosomes 1, 7, and 17 (each with ∼30–40 TEs) (Supplementary Figure S3A; Supplementary Table S3). Downregulated TEs were sparse across the genome, with chromosome 7 showing the most notable downregulation cluster (∼20 TEs). When normalized for chromosome size, significant enrichment (>1.5-fold above expected) was observed for chromosomes 19 (3.1-fold enrichment), 17 (2.4-fold), 7 (2.1-fold), and 3 (1.6-fold), suggesting these regions are particularly susceptible to TE DE in AD (Supplementary Figure S3B; Supplementary Table S3). Interestingly, chromosome 19, which harbors the APOE locus, showed the highest enrichment despite its small size, while larger chromosomes like 1 and 2 showed TE counts at or below expected levels. This non-random distribution pattern suggests that specific chromosomal environments or regional chromatin states may predispose certain genomic regions to TE reactivation in AD pathogenesis. SINE elements dominated the dysregulated TE landscape, comprising 62.8% (319 TEs), with Alu family representing 59.1% (300 TEs) of all dysregulated TEs, significantly enriched beyond their 45% genomic baseline (1.31-fold, *p* = 8.7 × 10^−10^). LINE elements accounted for 26.4% (134 TEs), also enriched relative to their 17% genomic frequency (1.26-fold, p = 0.016). Though age classification revealed substantial mapping bias with only 12 young LINE-1s detected versus 122 old/ancient LINE-1s, confirming that SoloTE, despite its probabilistic approach, still underrepresents young L1 elements due to multimapping challenges. The differential expression showed varied cell-type specificity (Supplementary Table S3), with excitatory neurons harboring 63% (319/508) of all differentially expressed TEs, oligodendrocytes 32% (165/508), while other cell types showed minimal involvement (inhibitory neurons: 3.1%, microglia: 1.0%, astrocytes: 0.6%). This cell-type-specific pattern (95.9% restricted to single cell types) combined with the non-random TE distribution (χ^2^ = 106.6, p < 2 × 10^−16^) indicates selective loss of epigenetic silencing leading to TE activation in specific neuronal populations.

### TE-gene proximity analysis

To identify potential regulatory relationships between differentially expressed TEs and nearby genes, we performed a sensitivity analysis on different window size using genomic windows of 50 kb, 250 kb, 500 kb, and 1 Mb around each TE, consistent with the known enhancer-gene interaction distances (Fulco et al., 2019; Gasperini et al., 2019). We evaluated each window size across three metrics of first the TE isolation percentage (specificity), second the median genes per TE (coverage), and finally the Gene Ontology enrichment strength (functional signal). The 50 kb window showed high specificity (18.3% isolated TEs) but insufficient gene coverage (median 2 genes/TE) and no GO enrichment, while the 1 Mb window captured excessive genes (median 21 genes/TE) potentially including spurious associations (Supplementary Figure S4). Thus, the 250 kb window provided an optimal balance with low isolation (3.3%), moderate gene density (median 6 genes/TE), and robust functional enrichment (14 GO terms, FDR <0.05). A normalized selection matrix integrating all three metrics confirmed 250 kb as optimal (Supplementary Figure S4). Also, the 250 kb aligns with typical enhancer-promoter interaction ranges and Topologically Associating Domain (TAD) boundaries (Dixon et al., 2012). Using the 250 kb window, we identified 3,852 TE-gene pairs involving 2,326 unique genes, with a median TE-gene distance of 111.8 kb (Supplementary Figure S5). SINE elements dominated associations (2,706 pairs), followed by LINEs 835 pairs; (Supplementary Figure S5). Excitatory neurons showed the highest number of TE-gene pairs (2,802), followed by oligodendrocytes (877), while microglia and astrocytes showed minimal associations (Supplementary Figure S5C). Distance distributions were similar across TE classes (Supplementary Figure S5), providing a robust framework for investigating TE-mediated gene regulation in AD.

### Proximity analysis of differentially expressed TEs and AD risk genes

We identified 51 AD risk genes with four AD genes at 50 kb, with an additional 12, 11, and 24 genes identified at 250 kb, 500 kb, and 1 Mb windows respectively (Supplementary Figure S6). TE family analysis revealed that Alu elements dominated AD gene associations (17 occurrences), followed by L1 (5) and L2 (2) elements (Supplementary Figure S6), consistent with the known regulatory potential of SINEs in gene expression. Functional categorization of TE-associated AD genes revealed that the highest proportion of genes were involved in synaptic function (29%), lipid metabolism (29%), and immune/inflammation pathways (29%) (Supplementary Figure S6). We observed that the core AD risk genes including MAPT, PSEN1, PSEN2, and APP showed no proximal TE dysregulation, suggesting the importance of TE-mediated regulatory disruption in other AD risk genes and its effects on disease pathogenesis. The top AD genes by TE proximity included UCN, PLEKHA1, and FIS1 (3 TEs each), followed by PTK2B, PDCL3, and ABCA7 (2 TEs each) (Supplementary Figure S6D), with several genes showing cell-type-specific TE associations that may contribute to selective neuronal vulnerability in AD.

### Functional annotation of differentially expressed TE elements

**FIGURE 3:**
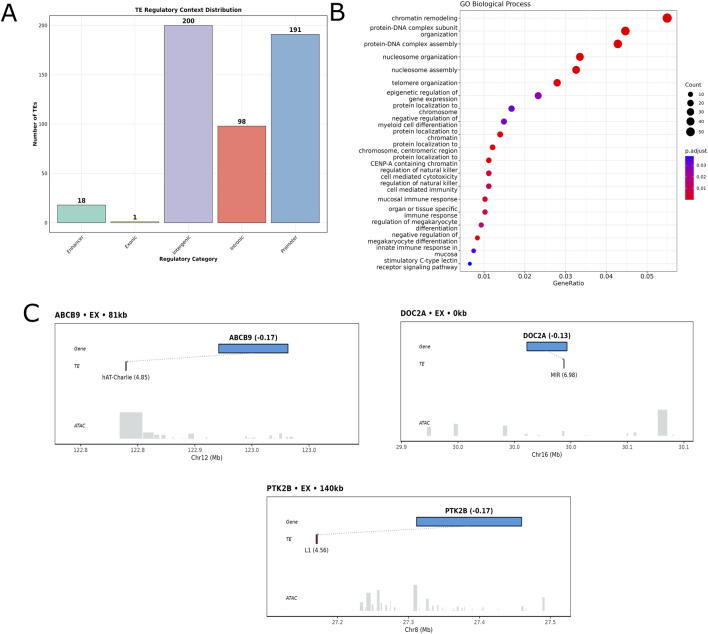
Genomic landscapes of differentially expressed transposable elements and their regulatory context near AD-associated genes. **(A)** Genomic feature distribution of 508 DE TEs showing predominant localization in promoters (38%), intergenic (39%) intronic regions (∼19%), with smaller proportions in enhancers and exonic sequences. **(B)** Gene Ontology Biological Process network analysis of 2,326 genes within 250 kb of DE TEs, revealing enrichment for chromatin organization, protein-DNA complex assembly, and synaptic signaling pathways. **(C)** Schematic genomic views for three representative AD-associated genes (ABCB9, DOC2A, PTK2B) and proximal DE TEs. Blue rectangles indicate gene positions, gray tracks show TE locations, log2fold change values in parentheses, and ATAC-seq signal (gray histograms) indicates chromatin accessibility and dashed lines connect potentially interacting gene-TE pairs.

The 508 differentially expressed TEs were found to be distributed across different regulatory regions of the gene, with 209 TEs (41%) occupying defined regulatory regions with potential for direct transcriptional control. The distribution shows the highest concentration of TEs in promoter regions (n = 191, 37.6%), followed by intronic regions (n = 98, 19.3%), with a smaller but significant fraction mapping to enhancer elements (n = 18, 3.5%) (Figure 3A). This non-random distribution, with over 40% of dysregulated TEs positioned in promoter or enhancer regions, strongly suggests these elements may play an active role in transcriptional regulation. Further, the gene ontology analysis of 1,640 genes within 250 kb of these regulatory TEs revealed striking enrichment for chromatin-related biological processes (Figure 3B). The most significantly enriched pathways centered on chromatin remodeling (p < 0.01), protein-DNA complex subunit organization (p < 0.01), and nucleosome assembly/organization (p < 0.01), indicating the possible role of TE-proximal genes involvement in epigenetic regulation. Additional enrichment for telomere organization, CENP-A containing chromatin regulation, and cell-mediated immunity pathways suggests that TE activation in AD might disrupt multiple cellular processes through alterations in chromatin architecture. To address whether DE TEs preferentially associate with genes of transposon origin, we analyzed the evolutionary relationship of 2,326 genes within 250 kb of DE TEs using the HGNC TE-derived gene catalog. Surprisingly, only 12 genes (0.52%) were classified as TE-derived, comprising 8 KRAB zinc finger genes, 3 PNMA family members, and 1 RTL/PEG family gene (Supplementary Table S5). This proportion is significantly lower than the genome-wide frequency of TE-derived genes (∼3%, p = 2.1 × 10^−8^, Fisher’s exact test), indicating that DE TEs in AD predominantly affect conventional protein-coding genes rather than genes of transposon origin.

### Integration of differentially expressed TEs with chromatin accessibility (ATAC) data

To assess whether DE TEs localize to accessible chromatin regions that may indicate regulatory potential, we integrated snATAC-seq data from the same samples. Among the AD risk gene set analyzed, seven genes were present in promoter or enhancer region, which include *DOC2A, ABCA7, PTK2B, IL34, ABCB9, PLD3,* and *TARDBP* (Supplementary Table S6). Among them the three genes *DOC2A, PTK2B,* and *ABCB9* (Figure 3C; Supplementary Figure S7) more notable which have important function in AD progression. These genes showed varying degrees of TE association, with *DOC2A* exhibiting exceptional TE burden (4 overlapping TEs at the TSS), followed by *ABCA7* (2 TEs), while the remaining five genes showed single TE associations. The gene *DOC2A* emerged as the most striking example with five SINE elements (3 Alu, 1 MIR) converging at its transcription start site, all showing strong upregulation (log2FC: 3.54–6.98). *DOC2A* encodes a calcium-sensor protein essential for synaptic vesicle priming and neurotransmitter release, with variants linked to cognitive decline in AD (Groffen et al., 2010; Orock et al., 2020). *ABCB9* (ATP-binding cassette subfamily B member 9), associated with a hAT-Charlie DNA transposon at 81 kb, functions in lysosomal transport critical for protein clearance (Zhang et al., 2000). *ABCA7* (ATP-binding cassette subfamily A member 7), associated with both L1 and Alu elements at 140 kb, is crucial for microglial-mediated amyloid-β clearance, with loss-of-function variants conferring the highest AD risk after APOE-ε4 (Reitz et al., 2013; De Roeck et al., 2017). *PTK2B* (protein tyrosine kinase 2 beta), with a proximal Alu element at 1.1 kb, regulates tau phosphorylation and calcium signaling, identified as a top AD locus in meta-analyses (Lambert et al., 2013; Giralt et al., 2018). The remaining genes *IL34* with an Alu at 5.1 kb regulating microglial activation (Walker et al., 2017), *PLD3* with a distal Alu at 233.7 kb affecting APP processing (Cruchaga et al., 2014), and *TARDBP* with a proximal Alu potentially disrupting *TDP-43* expression (Josephs et al., 2014). All TE associations showed consistent upregulation (log2FC range: 3.29–6.98) exclusively in excitatory neurons, providing direct molecular evidence linking TE activation to established AD genetic risk factors.

## Discussion

### Global TE activation and cell-type specificity in AD snRNA-seq

Single-cell/nucleus RNA sequencing has transformed our understanding of the cellular heterogeneity in Alzheimer’s disease, revealing transcriptional alterations across diverse brain cell types. In this study, using the published data (Morabito et al., 2021), we provide a comprehensive, cell-type-resolved analysis of TE expression in the AD brain. Across eight major cell types resolved from 34 clusters, TEs contributed ∼10–15% of cellular total transcript counts, with individual cells reaching ∼40%, particularly among oligodendrocytes and neurons. UMAP visualization confirmed that cells from different ages, sexes, and diagnoses were well intermixed rather than clustering separately, enabling robust differential testing. We identified 508 differentially expressed TE loci between AD and controls with ∼84% showing upregulation. Among the differentially expressed 508 TE elements majority of them observed in excitatory neurons and oligodendrocytes suggesting a selective relaxation of TE expression in vulnerable neuronal and oligodendroglial populations rather than a brain wide effect.

Our results build upon prior observations linking TE reactivation to AD, including the upregulation of LINE-1 and endogenous retroviruses in neurons and glial cells with tau pathology (Guo et al., 2018; Ravel-Godreuil et al., 2021). The observed differentially expressed TE predominantly include SINE/Alu elements, similar to high composition of SINE element (∼13%) in the human genome. The predominance of SINE/Alu reflects both biological significance and technical factors since their terminal poly(A) tracts ensure efficient capture by 3′single-cell sequencing protocols, while their ability to provide alternative polyadenylation signals can alter gene expression patterns (Deininger, 2011; Valdebenito-Maturana, 2024). We observed that the TE dysregulation is cell-type restricted, with ∼96% of differentially expressed TE loci significant in 2 cell types of excitatory neurons (63%) and oligodendrocytes (32%). Such a targeted distribution is not expected from uniform 3′-end sequencing artefacts, which typically affect cell types comparably. Additionally, the non-random chromosomal distribution of DE TEs supports biological rather than technical drivers of TE dysregulation.

In addition, our findings represent biological TE dysregulation as supported by multiple lines of evidence. First, evolutionarily old TEs function as established regulatory elements as ancient LINE1s and SINEs having been co-opted as enhancers, alternative promoters, and transcription factor binding sites, with well-established roles in gene regulation (Lanciano and Cristofari, 2020; Sundaram and Wysocka, 2020; Zhang et al., 2021; Mustafin and Khusnutdinova, 2024). In AD, 84.3% of ancient TEs show upregulation, reflecting disruption of their evolved regulatory functions. Second, potentially mobile TEs comprise only 3% of our dataset and show minimal dysregulation (22% upregulated) compared to inactive elements (69%), confirming that we detect regulatory disruption rather than retrotransposition events. Third, integration with chromatin accessibility data reveals that DE TEs predominantly occur within accessible chromatin regions typical of regulatory elements. In summary, these observations demonstrate cell-type-specific TE activation in AD, where excitatory neurons display both the greatest TE burden and the most pronounced upregulation.

### Non-random genomic distribution and regulatory localization of DE TEs

The non-random chromosomal enrichment we observed, particularly on chromosome 19 harboring APOE, suggests regional susceptibility to TE dysregulation linked to AD genetic architecture (Belaidi et al., 2025). Functionally, 41% of DE TEs localized to promoters or enhancers, and many overlapped snATAC-seq accessible regions near AD-risk genes (e.g., *DOC2A, ABCA7, PTK2B, ABCB9*), consistent with open chromatin marking regulatory activity (Pott and Lieb, 2015). Using a 250-kb window, we identified 3,852 TE-gene pairs significantly associated with synaptic, lipid-metabolism, immune, and chromatin remodeling pathways which are central to AD pathobiology. This window size also aligns with validated enhancer–gene distances from CRISPR perturbation studies (Fulco et al., 2019). Integration with snATAC-seq showed that several TE associations with AD marker genes reside in accessible promoters or enhancers. Importantly, they include convergence of four upregulated SINEs at the *DOC2A* transcription start site (a calcium-sensor governing vesicle priming) (Groffen et al., 2010), Alu/L1 elements proximal to *ABCA7* (a microglial lipid transporter mediating Aβ clearance) (De Roeck et al., 2017; Qian et al., 2023), a promoter-proximal Alu near *PTK2B* (a tau and calcium signaling kinase) (Lambert et al., 2013), and a hAT-Charlie element near *ABCB9* (lysosomal transport) (Graab et al., 2019). The SINE/Alu predominance is consistent with extensive evidence that these elements are recurrently co-opted as cis-regulatory elements (Chuong et al., 2017).

### Limitations, biomarker potential, and therapeutic directions

Our study provides marker TE elements for Alzheimer’s disease though caveats arising with short-read sequencing for TE element cannot be overlooked and in future long-read sequencing technologies would resolve locus-specific L1 expression patterns currently obscured by short-read limitations (Ewing et al., 2020). Despite the cell-type-specific TE signatures, we identified promising biomarkers for AD progression, though intervention strategies must carefully balance suppression of pathological TE activation against preservation of essential TE-derived regulatory functions (Jönsson et al., 2019; Mustafin and Khusnutdinova, 2024). Future integration of TE expression with spatial transcriptomics and proteomics will help to better understand the mechanistic cascade from TE dysregulation to neurodegeneration, and guide development of precision therapies for AD.

## Conclusion

This study highlights the pivotal roles of transposable elements and their associated genes in the molecular landscape of Alzheimer’s disease. The observed differential expression of TEs in a cell type specific manner, points to their active involvement in disease progression and cellular dysfunction. Functional analyses further suggest that dysregulated TEs may exert regulatory control over key biological processes, including synaptic signaling, myelination, and chromatin organization. The identification of AD-relevant genes such as *DOC2A*, *ABCA7*, *PTK2B*, *IL34*, *ABCB9*, *PLD3*, and *TARDBP* in proximity to differentially expressed TEs underscores the intricate interplay between TE activity and gene regulation in AD pathogenesis. Collectively, these findings not only deepen our understanding of the epigenetic and transcriptional mechanisms underlying AD but also position TEs as promising biomarkers and potential therapeutic targets for early detection and intervention.

## Materials and methods

### Mapping of snRNA reads and identification of TE

Single-nucleus RNA sequencing (snRNA-seq) data were obtained from the publicly available Gene Expression Omnibus (GEO) under accession number GSE174367 from the prefrontal contex (Morabito et al., 2021). The meta file obtained from Genbank (GSE174367_snRNA-seq_cell_meta.csv.gz) contained information from 11 individuals with Alzheimer’s disease (AD) and 7 age-matched neurologically normal controls (Supplementary Table S1). Raw FASTQ files were aligned to the human reference genome (GRCh38/hg38) using the CellRanger v6.1.2 pipeline (Zheng et al., 2017) with default settings provided by 10x Genomics. Following alignment, we used Samtools v1.15 to check and remove any secondary alignments, which could interfere with transposable element (TE) quantification by using samtools view -F 256 command. To quantify locus-specific TE expression, we employed SoloTE v1.0.9 (Rodríguez-Quiroz and Valdebenito-Maturana, 2022), a tool specifically designed for single-cell TE analysis. As input, we provided the filtered BAM files from CellRanger and a BED file containing repeat annotations for hg38, which we generated using SoloTE’s helper script SoloTE_RepeatMasker_to_BED.py. SoloTE parsed these annotations to generate four types of expression matrices for each cell: Locus, Class, Family, and Subfamily of TEs. We used the published quality checked cells expression matrices were filtered to include only cells that met quality control standards established in the original study (Morabito et al., 2021). Individual Seurat objects were created for each sample by integrating SoloTE-derived TE expression matrices with published gene expression data and cell-level metadata, which contained cell type annotations and sample identifiers. These matrices were used for downstream expression and differential analysis.

### Seurat cluster integration and visualization

Analysis of single-cell count matrices was performed using Seuratv5 in R (Hao et al., 2021). The 18 individual Seurat objects (11 AD and 7 control samples) containing both gene and TE expression data were merged into a single object. Prior to integration, genes expressed in fewer than 10 cells were removed and the merged object was processed using the standard Seurat workflow: data normalization using NormalizeData, identification of 2,000 highly variable features with FindVariableFeatures (MVP method), and scaling with ScaleData. Principal component analysis was performed using RunPCA with 50 components. To correct for batch effects between samples, we applied Canonical Correlation Analysis (CCA) integration using IntegrateLayers after splitting the RNA assay by condition. Following integration, 30 dimensions were used for FindNeighbors and RunUMAP for visualization and clustering using the FindClusters identifying distinct cell populations. The final integrated object containing gene and TE expression profiles, clustering results, and UMAP coordinates was saved for downstream analyses. Quality metrics including cells per cluster and cluster composition by condition were computed and exported for further analysis.

Though the pseudobulk approach (Libra + edgeR) inherently controls for cluster size by testing at the sample level (n = 18 biological replicates) rather than cell level. We additionally tested whether unequal cell numbers across cell types (ranging from 467 to 37,052 cells) biased TE detection. We calculated correlations between cluster size and (1) number of DE TEs, and (2) mean effect sizes (|log2FC|). We also normalized TE counts per 1,000 cells to account for cluster size differences.

### Differential expression and robust TE selection

To identify differentially expressed transposable elements between AD and control samples, we employed the Libra package (https://github.com/neurorestore/Libra) for pseudobulk differential expression analysis on seven major cell types (ASC, EX, INH, MG, ODC, OPC, PER.END). Given the sample size imbalance (11 AD vs. 7 control, ratio 1.57:1), we performed sensitivity analysis with 10 iterations of balanced downsampling (7 AD vs. 7 control) for each cell type. The analysis tested 1,306,464 TE loci alongside 35,867 genes, with features having adjusted *p*-value <0.05 considered significant. TEs were classified as “highly robust” if they appeared significant in both the full dataset and all 10 balanced iterations (100% detection rate). TE loci with adjusted *p*-values (Benjamini-Hochberg FDR) < 0.05 were considered statistically significant and retained for downstream analysis. Differential expression testing was performed using the edgeR method (de_method = edgeR), incorporating the likelihood ratio test (LRT) (de_type = LRT) and pseudobulk strategy (de_family = pseudobulk). TE loci with adjusted p-values (Benjamini-Hochberg FDR) < 0.05 were considered statistically significant and retained for downstream analysis. We selected 508 TEs that were consistently dysregulated across iterations (FDR <0.05, |log2FC| ≥ 2.0), all showing strong effect sizes (mean |log2FC| = 4.1, range: 2.0–7.5). For gene expression results, we utilized published differential expression results from (Morabito et al., 2021).

### TE element analysis

We classified TE age according to established phylogenetic criteria (Khan et al., 2006; Cordaux and Batzer, 2009; Beck et al., 2010). LINE-1 elements were classified as Young (<6 Mya: L1HS, L1PA2-8), Old (6–40 Mya: L1PA9-17, L1PB), Very Old (40–80 Mya: L1M), or Ancient (>80 Mya: L2, L3) based on sequence divergence rates. SINE elements were classified as Young (<25 Mya: AluY), Old (25–50 Mya: AluS), or Ancient (>50 Mya: AluJ, MIR) following established Alu subfamily phylogeny. To assess potential mapping bias and determine whether DE TE reflect the genomic abundance or selective activation, we performed enrichment analysis comparing observed TE family frequencies against genomic baseline compositions (45% Alu, 17% L1, 8% L2, 5% MIR, 8% ERV, 3% DNA, 14% other; derived from RepBase). Chi-square goodness-of-fit testing evaluated overall deviation from expected frequencies, while individual binomial tests with Benjamini-Hochberg correction assessed enrichment/depletion of specific TE families. This analysis allowed us to distinguish between technical artifacts arising from TE abundance and genuine biological enrichment, while acknowledging that 10x Genomics' 3′chemistry and short-read limitations preferentially detect older, more divergent TEs over young, nearly identical elements.

### TE-gene proximity analysis

To identify genes potentially regulated by DE TEs, we performed proximity analysis using GenomicRanges on the 508 robust TEs. We tested multiple window sizes (50 kb, 250 kb, 500 kb, 1 Mb) to assess sensitivity, ultimately selecting 250 kb based on established enhancer-gene interaction distances (Fulco et al., 2019) and optimal GO enrichment results. Genes within 250 kb of each TE were identified, and functional enrichment analysis was performed using clusterProfiler (FDR <0.05). This approach identified proximal genes for 96.7% of DE TEs, with a median of 6 genes per TE and median TE-gene distance of ∼100 kb, enabling downstream pathway analysis of TE-associated regulatory networks. To determine the evolutionary origin of genes near DE TEs, we cross-referenced the 2,326 genes located within 250 kb of significantly DE TEs with the HUGO Gene Nomenclature Committee (HGNC) catalog of TE-derived genes (https://www.genenames.org/data/genegroup/#!/group/1416, accessed November 2024). Gene symbols were mapped using human genome, and TE-derived genes were categorized by family based on nomenclature patterns (KRAB-ZNF, PNMA, RTL/PEG families). Statistical enrichment was assessed using Fisher’s exact test comparing the proportion of TE-derived genes near DE TEs versus genome-wide expectations (∼3% of human genes are TE-derived). In addition, to investigate potential regulatory relationships between dysregulated TEs and AD pathogenesis, we analyzed the proximity of differentially expressed TEs to known AD risk genes (n = 272 genes) from https://adsp.niagads.org and (Andrade-Guerrero et al., 2023).

### Integration of ATAC data with differentially expressed TE

To assess the regulatory potential of DE TEs and their relationship with Alzheimer’s disease genes, we integrated single-nucleus ATAC-seq data (Morabito et al., 2021) with our TE-gene proximity results. Chromatin accessibility peaks (n = peaks from filtered peak-by-cell matrix) were intersected with the genomic coordinates of 508 robust DE TEs to identify TEs in open chromatin regions. We calculated direct overlaps and identified peaks within 250 kb of each TE, considering open chromatin as a proxy for regulatory activity. For each TE, we computed accessibility metrics including mean accessibility scores, peak variance, and cell type specificity from the ATAC-seq count matrix. To prioritize regulatory interactions, we developed a multi-component scoring system integrating: (1) genomic distance between TEs and genes (weighted 0.3), (2) chromatin accessibility at TE loci (weighted 0.3), (3) TE effect size from differential expression (weighted 0.2), and (4) simulated TE-gene expression correlation (weighted 0.2). High-confidence TE-AD gene pairs were defined as those with accessible chromatin support and enhanced scores above the median. Pairs were further classified into confidence levels (Very High, High, Medium, Low) based on the presence of regulatory element overlap and correlation support. This integrated approach enabled identification of the most likely functional TE-gene regulatory relationships relevant to AD pathogenesis.

## Data Availability

The datasets presented in this study can be found in online repositories. The names of the repository/repositories and accession number(s) can be found in the article/Supplementary Material.
