# Supplementary material for: Cellular insights into transposable elements in Alzheimer’s disease
Source: Front Mol Biosci. 2026 Jan 7;12:1642599. doi: 10.3389/fmolb.2025.1642599 (PMC12819740; doi:10.3389/fmolb.2025.1642599)
Supplement: Supplementary file 1 [file DataSheet1.pdf]

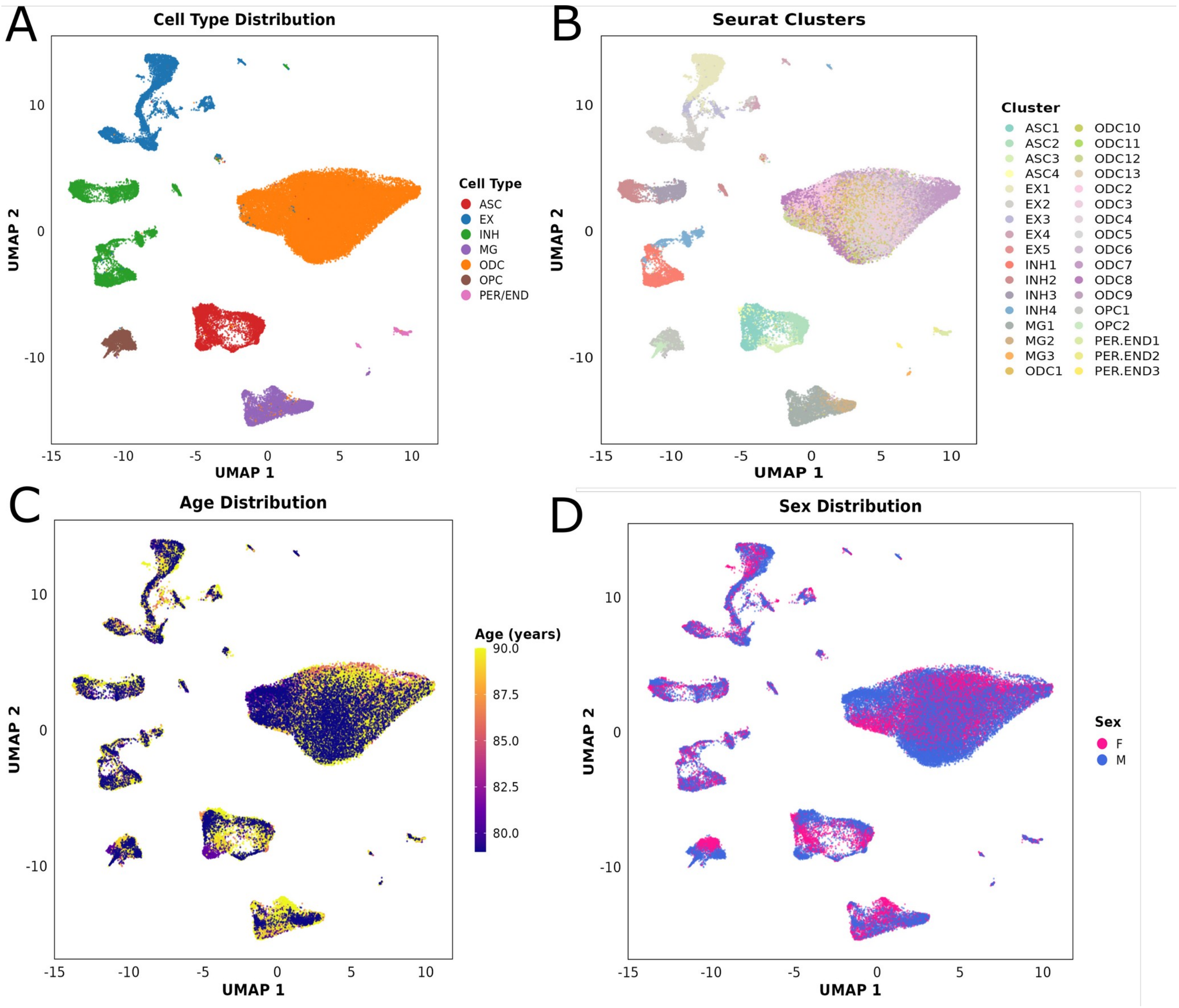

Figure S1. UMAP visualization of single-nucleus RNA-seq data by cell type, detailed cell type, age, and sex (A) UMAP of 61,472 nuclei colored by cell type, including neurons, astrocytes, oligodendrocytes, microglia, endothelial cells, and pericytes/endothelial cells (PER/END). (B) UMAP projections of 61,472 nuclei colored by detailed subcluster annotations within major brain cell types. (C) and (D) UMAP colored by Age and donor sex (male/female) showing no age and sex-specific segregation.

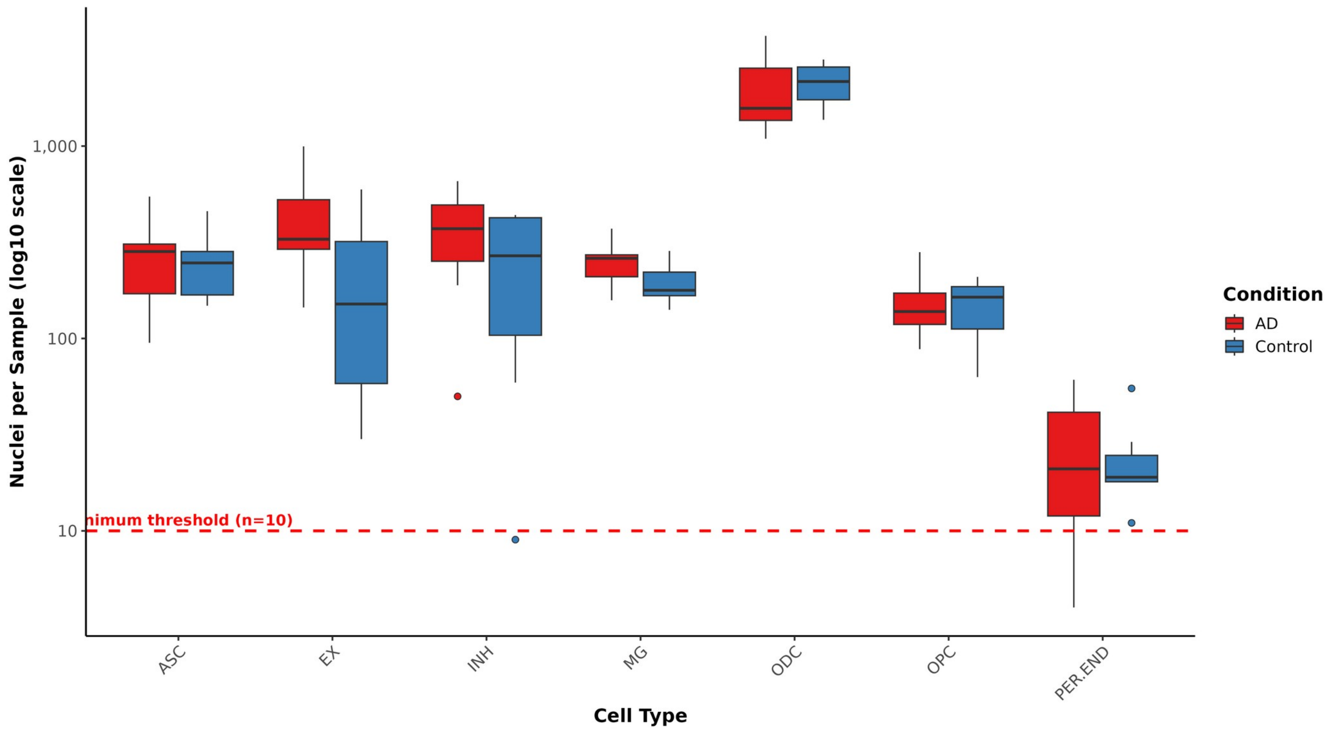

Fig S2: Distribution of Nuclei Counts per Sample (Major Cell Types) Box plots showing nuclei count distributions per sample for major cell types (≥10 samples) by condition (AD = red, Control = blue), displayed on a log10 scale. Horizontal dashed red line indicates the minimum threshold (n=10) for pseudobulk analysis inclusion. ODC shows the highest counts (~1000-2000), while PER.END shows the lowest (~10-50). Most cell types show comparable distributions between AD and control conditions, with some samples falling below the threshold particularly in rare cell types.

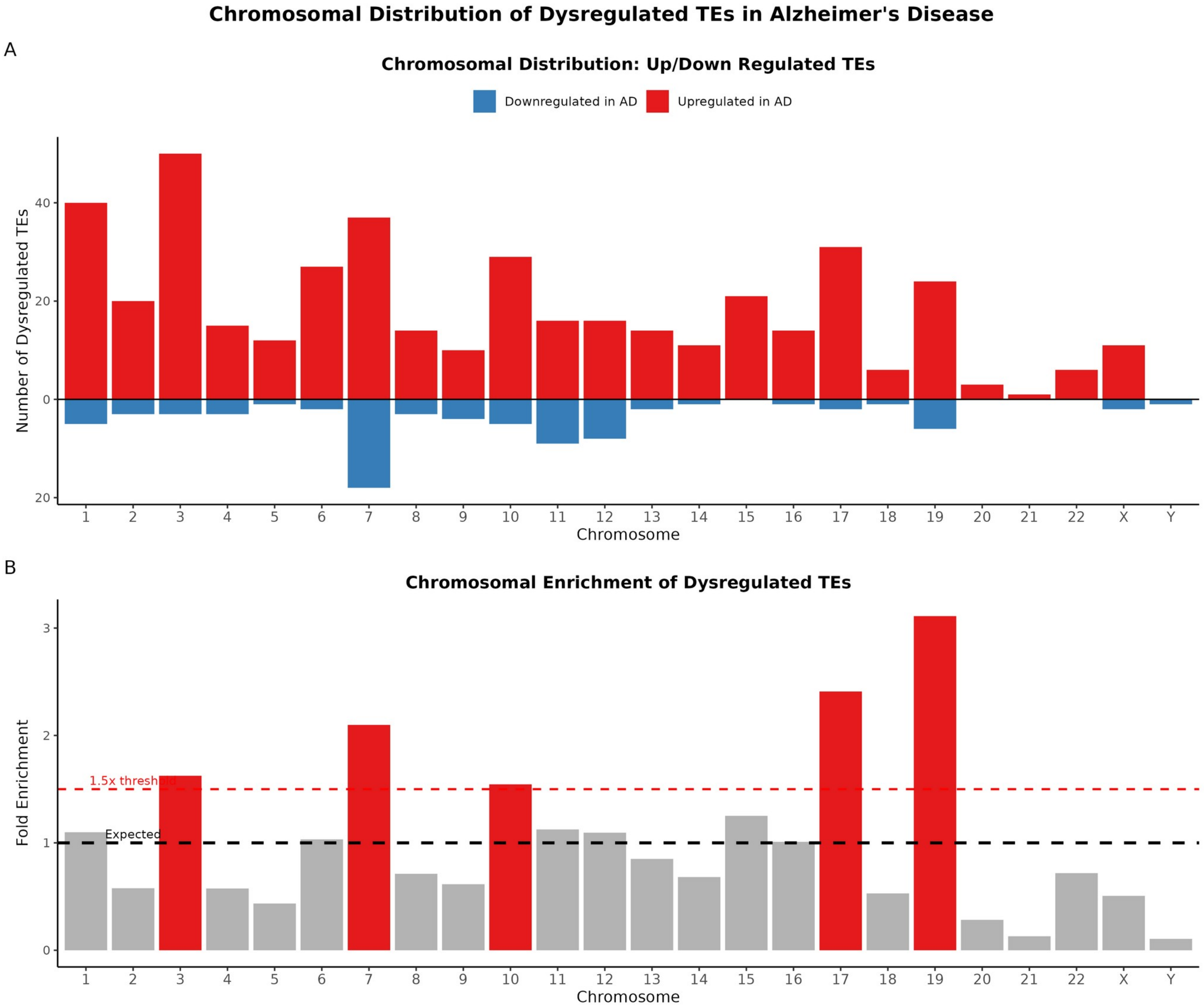

Figure S3: Chromosomal Distribution of DE Transposable Elements in Alzheimer's Disease (A) Stacked bar chart showing counts of upregulated (red, above axis) and downregulated (blue, below axis) TEs per chromosome. TE DE is predominantly upregulation across all chromosomes, with chromosome 3 showing the highest number (~50 TEs), followed by chromosomes 1, 7, and 17. Downregulation is rare and most prominent on chromosome 7 (~20 TEs). **(B)** Fold enrichment of dysregulated TEs relative to expected distribution (gray bars = expected; red bars = significantly enriched above 1.5× threshold). Chromosomes 19 (~3-fold), 17 (~2.4-fold), and 7 (~2.1-fold) show significant enrichment, indicating preferential TE DE in these genomic regions beyond what would be expected by chromosome size alone.

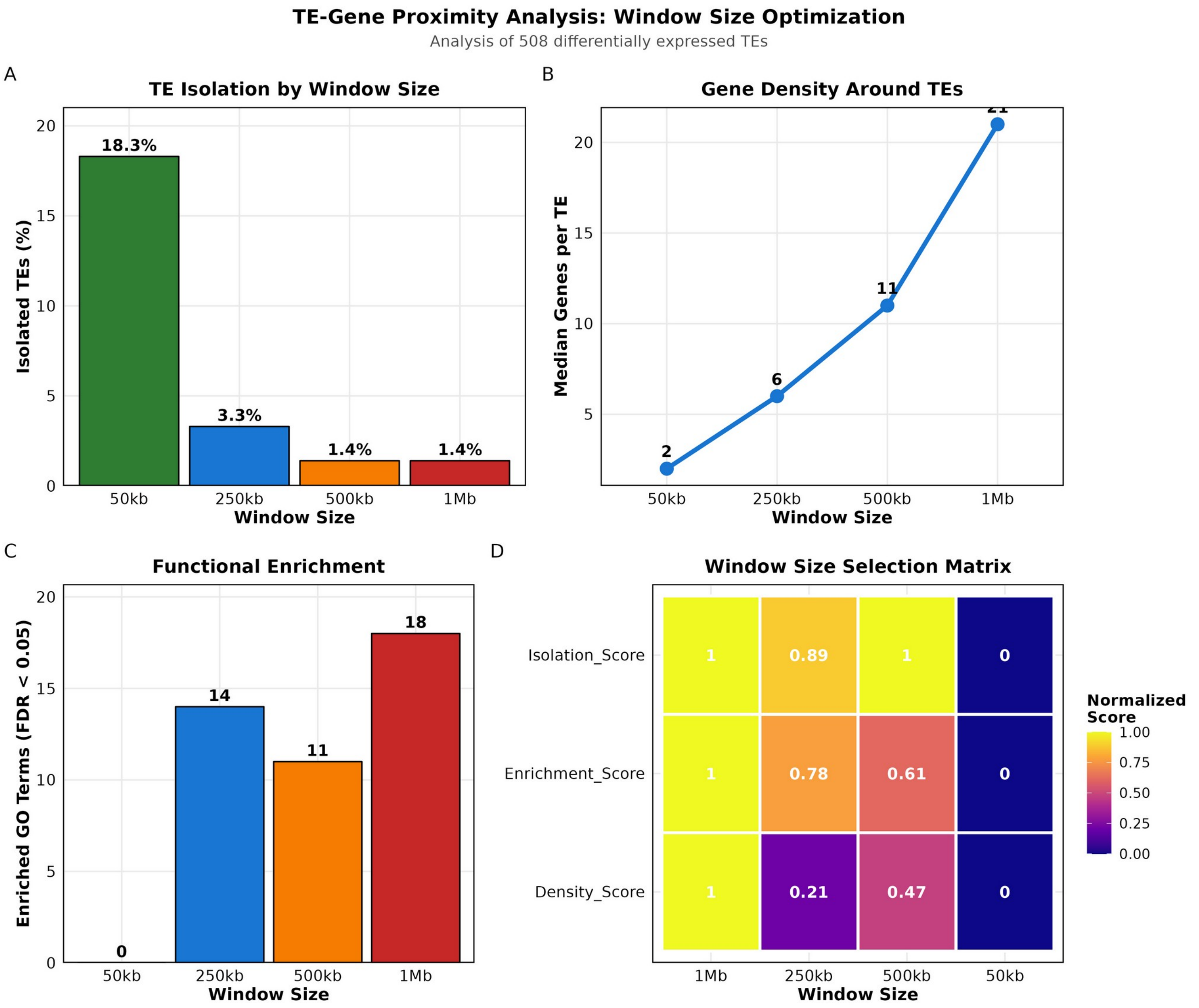

Figure S4. Window size optimization for TE-gene proximity analysis. (A) Percentage of isolated TEs (without nearby genes) decreases from 18.3% at 50kb to 1.4% at larger windows. (B) Median genes per TE increases from 2 at 50kb to 21 at 1Mb. (C) GO enrichment analysis reveals 14 significant terms at 250kb and 18 at 1Mb, with no enrichment at 50kb. (D) Normalized selection matrix integrating specificity (Isolation Score), coverage (Density Score), and functional enrichment (Enrichment Score) identifies 250kb as the optimal window, balancing all three metrics. This window size was selected for all downstream TE-gene association analyses and is consistent with typical enhancer-promoter interaction distances. Analysis based on 508 differentially expressed TEs (FDR < 0.05).

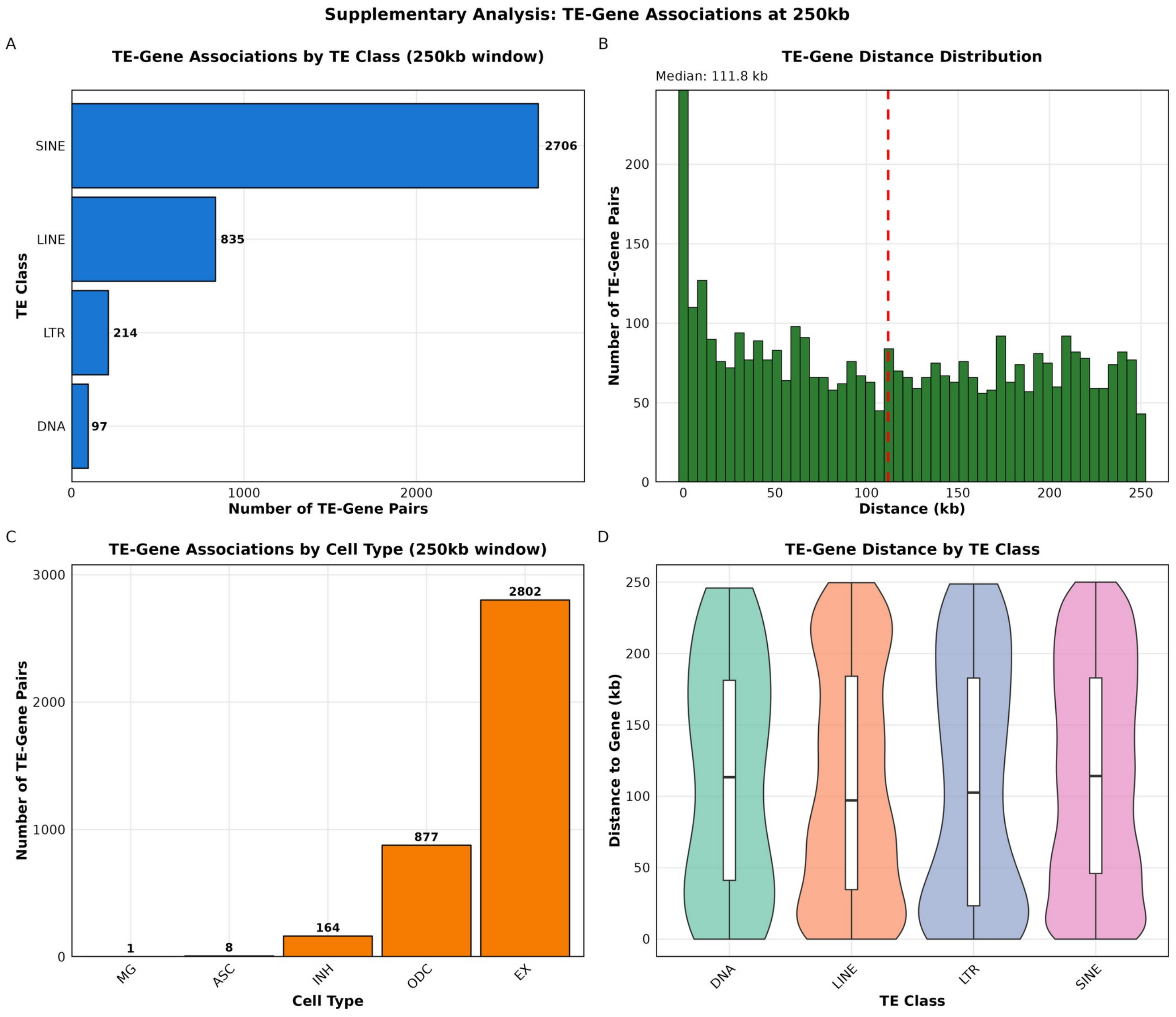

Figure S5. Characterization of TE-gene associations within 250kb genomic windows. (A) Distribution of TE-gene pairs by TE class, showing SINE predominance (2,706 pairs). **(B)** Distance distribution between TEs and nearby genes (median = 111.8 kb). **(C)** Cell-type-specific TE-gene associations, with excitatory neurons showing the highest number of pairs (2,802). **(D)** TE-gene distance distributions stratified by TE class, showing similar patterns across all major classes (median ~100-120 kb). Analysis based on 508 differentially expressed TEs; 250kb window selected based on optimization analysis (Figure [X]).

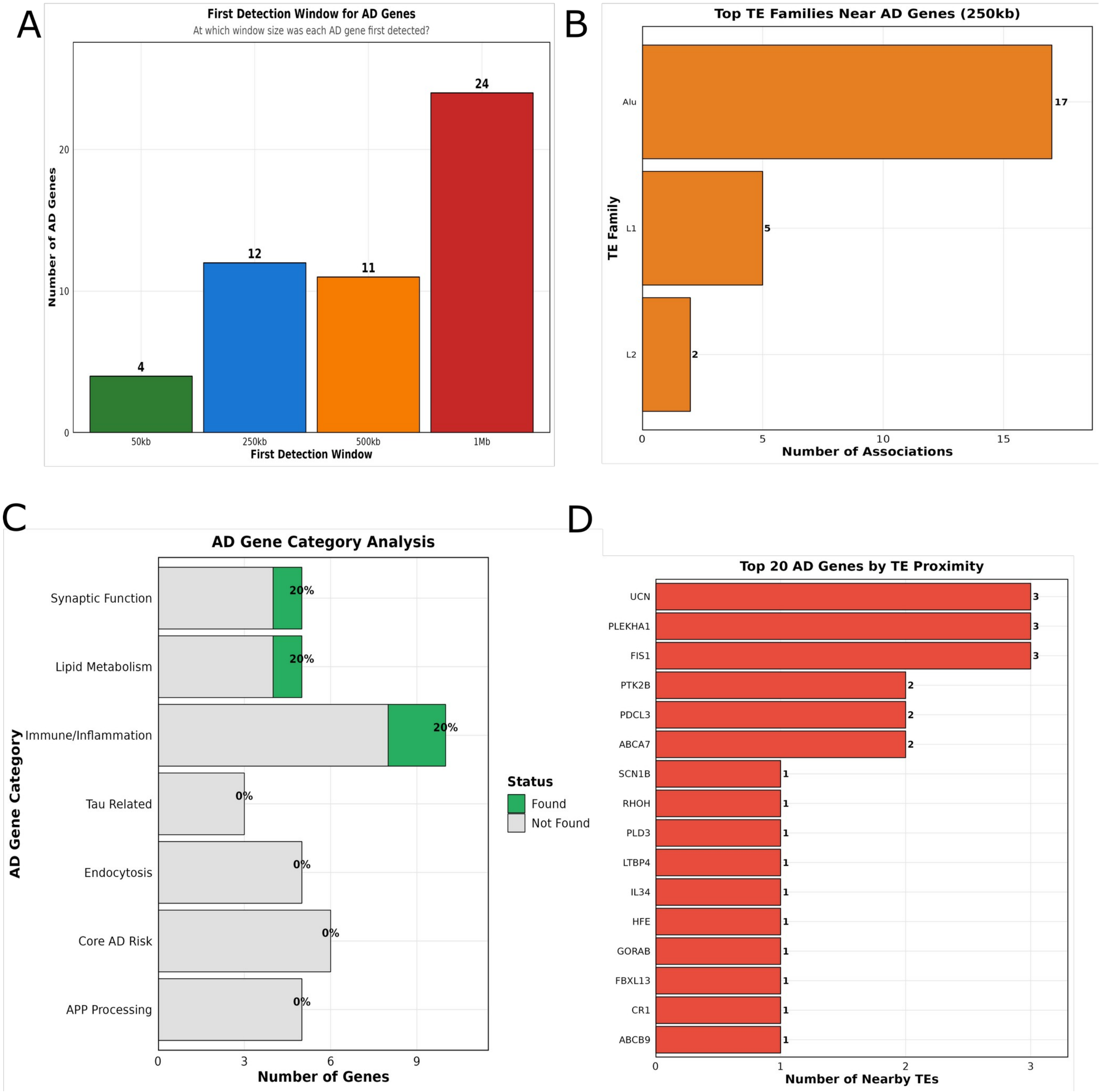

Figure S6. Transposable element associations with Alzheimer's disease risk genes. (A) Window size analysis showing 24 AD genes with TEs detected within 1Mb, with 250kb (blue) selected as optimal for capturing regulatory interactions while maintaining specificity. **(B)** TE family enrichment near AD genes showing Alu elements (17 associations) as the predominant family, followed by L1 (5) and L2 (2) elements. **(C)** Functional distribution of TE-associated AD genes revealing enrichment in synaptic function (29%), lipid metabolism (29%), and immune/inflammation (20%) pathways, while core AD genes (tau, APP processing) showed no TE associations.**(D)** Top 20 AD genes ranked by proximal TE count. UCN, PLEKHA1, and FIS1 each harbor 3 TEs; PTK2B, PDCL3, and ABCA7 have 2 TEs; remaining genes show single associations.

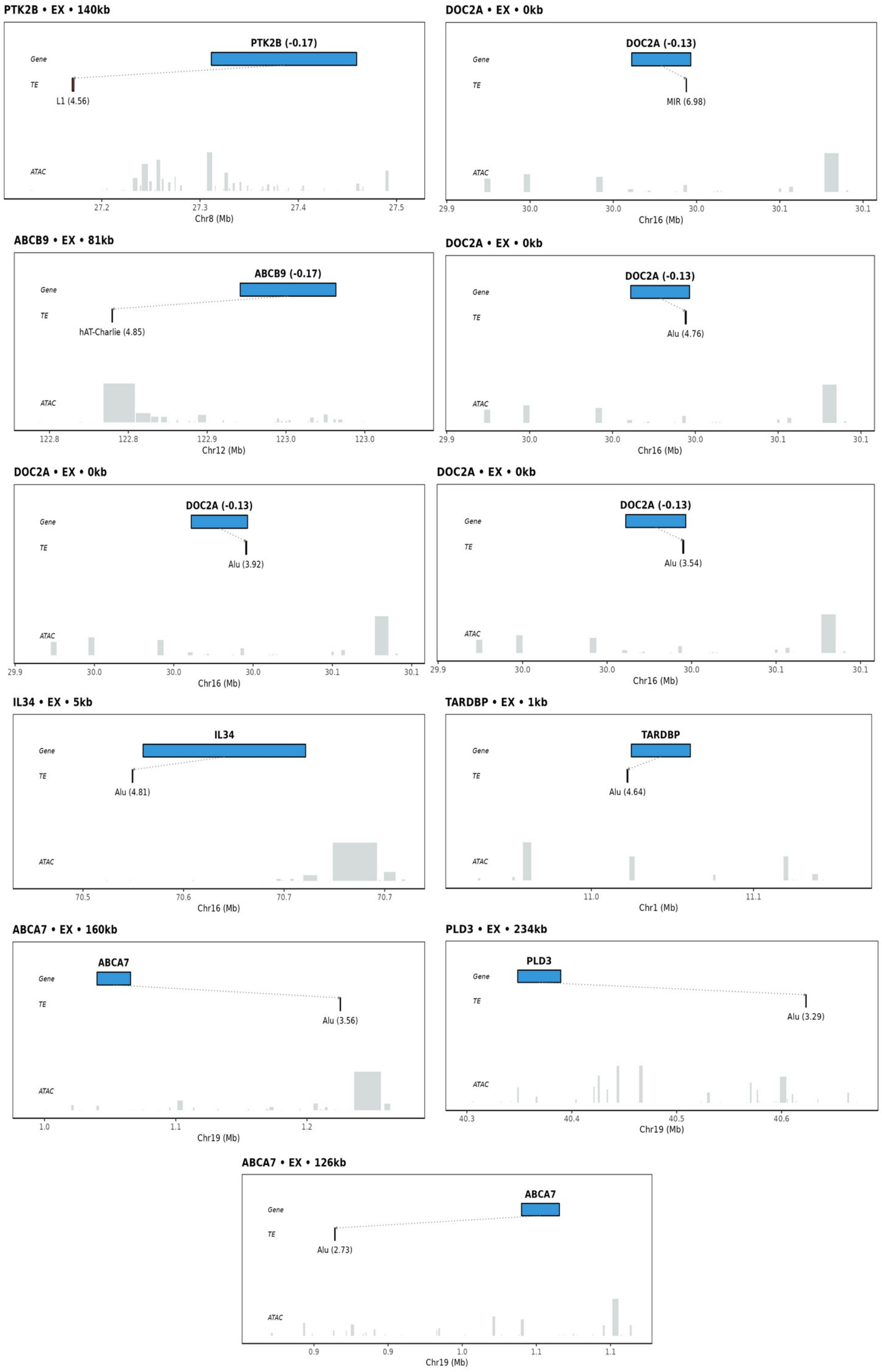

Figure S7. Schematic genomic views for all the identified representative AD-associated genes and proximal DE TEs. Blue rectangles indicate gene positions, gray tracks show TE locations, log2fold change values in parentheses, and ATAC-seq signal (gray histograms) indicates chromatin accessibility and dashed lines connect potentially interacting gene-TE pairs.
